# Complete mitochondrial genome of the Thai Red Junglefowl (*Gallus gallus*) and phylogenetic analysis

**DOI:** 10.24272/j.issn.2095-8137.2017.028

**Published:** 2018-03-07

**Authors:** Chatmongkon Suwannapoom, Ya-Jiang Wu, Xing Chen, Adeniyi C. Adeola, Jing Chen, Wen-Zhi Wang

**Affiliations:** 1 School of Agriculture and Natural Resources, University of Phayao, Phayao 56000, Thailand; 2 Southeast Asia Biodiversity Research Institute (CAS-SEABRI), Chinese Academy of Sciences, Yezin Nay Pyi Taw 05282, Myanmar; 3 State Key Laboratory for Conservation and Utilization of Bio-resource in Yunnan, Yunnan University, Kunming Yunnan 650091, China; 4 State Key Laboratory of Genetic Resources and Evolution, Kunming Institute of Zoology, Chinese Academy of Sciences, Kunming Yunnan 650223, China; 5 Wildlife Forensics Science Service Centre, Kunming Yunnan 650203, China; 6 Guizhou Academy of Testing and Analysis, Guizhou Academy of Sciences, Guiyang Guizhou 550002, China

**DEAR EDITOR**,

In this study, we sequenced the complete mitochondrial genome (mitogenome) of the Thai Red Junglefowl (RJF; *Gallus gallus*) using the next-generation sequencing (NGS) platform of the Ion Torrent PGM. Samples were taken from Mae Wang District, Chiang Mai Province, northern Thailand. Our data showed the complete mitogenome to be 16 785 bp in length, composed by 13 protein-coding genes, 22 tRNA genes, two rRNA genes, and one control region. The genome nucleotide composition was 30.3% A, 23.7% T, 32.5% C, and 13.5% G, resulting in a high percentage of A+T (50.4%). Phylogenetic analysis revealed that the mitogenome belonged to haplogroup X, whereas those of all domestic chickens belong to haplogroups A to G. This newly released mitogenome sequence will advance further evolutionary and population genetics study of the RJF and domestic chicken. The availability of the *G. gallus* mitogenome will also contribute to further conservation genetics research of a unique species, listed as ‘data deficient’ in Thailand.

The Red Junglefowl (RJF; *Gallus gallus*) is a major wild ancestor of the domestic chicken (Darwin, 1875; Liu et al., 2006; Miao et al., 2013). Early studies based on mitochondrial DNA (mtDNA) revealed that the Thai RJF has a close relationship with the domestic chicken (Fumihito et al., 1994; Fumihito et al., 1996), implying that Thailand is likely a domestication center of the chicken. To the best of our knowledge, no complete mtDNA genome (i.e., mitogenome) sequence of the Thai RJF has been reported. In this study, we collected a RJF sample from the Mae Wang District of Chiang Mai Province in northern Thailand (permission provided by the Thai Institute of Animals for Scientific Purpose Development (No. U1-01205-2558)). The complete mitochondrial genome was submitted to GenBank (accession No.: MG605671).

Genomic DNA was extracted from whole blood using the HiPure Tissue DNA Micro Kit (Magen, China). The PCR amplification, library construction, and next-generation sequencing were in accordance with our earlier study (Chen et al., 2016). We used a *de novo* long fragment PCR and NGS strategy to obtain high quality mtDNA reads and exclude NTMT pseudogenes. We followed caveats for quality control in mtDNA genomic studies of domestic animals (Shi et al., 2014). The generated sequence was aligned against a reference sequence AP003321 (Nishibori et al., 2005), with all variants then output. Using the Integrative Genomics Viewer (Thorvaldsdóttir et al., 2013), we checked the bam file exported by Torrent Suite 5.0.2 to confirm the scored variants.

Phylogenetic analysis was performed using complete mtDNA sequences of all major haplogroups and sub-haplogroups, as defined by Miao et al. (2013) and Peng et al. (2015). All mitogenomes were aligned by ClustalW, then analyzed by maximum parsimony (MP) in MEGA 7.0 with 1 000 bootstrap replicates (Tamura et al., 2011).

The complete mitogenome sequence of the Thai RJF (16 785 bp; GSA accession No.: PRJCA000287, GenBank accession No.: MG605671) had an overall base composition of 30.3% for A, 23.7% for T, 32.5% for C, and 13.5% for G, with high a A+T content of 54.0%. The mitogenome consisted of 13 protein-coding genes, 22 tRNA genes, two rRNA genes, and a displacement loop (D-loop). Most mitogenome genes were encoded on the heavy strand, except for eight tRNA genes and one protein-coding gene (*ND6*), which were encoded on the light strand. Some protein-coding genes shared the start and stop codons; for instance, all 13 genes began with ATG, except for *COX1*, which started with GTG, and of the remaining 12 protein-coding genes, nine (*ND1*, *COX2*, *ATPase8*, *ATPase6*, *ND3*, *ND4L*, *ND5*, cyt *b*, and *ND6*) shared the stop codon TAA, two (*COX3* and *ND4*) shard the stop codon “T– –”, and *ND2* used TAG. The lengths of the 12S rRNA and 16S rRNA genes were 976 bp and 1 622 bp, respectively.

As the RJF is a wild type of *Gallus gallus*, it differed from all major domestic haplogroups (A to G). We calculated the differences between the Thai RJF and randomly selected individuals with haplogroups A to G (Table 1). Compared with the common domestic haplogroups, the RJF had 40 different base pairs on average (range: 29–53). 

**Table 1:** Mitochondrial genome differences vs. Thai Red Junglefowl (PRJA000287)

Haplogroup	Sequence	Different base number (*n*)
A	GU261684	48
B	GU261714	45
C1	GU261679	53
D	GU261683	42
E	GU261712	29
F	GU261717	34
G	GU261676	31

**Figure 1:**
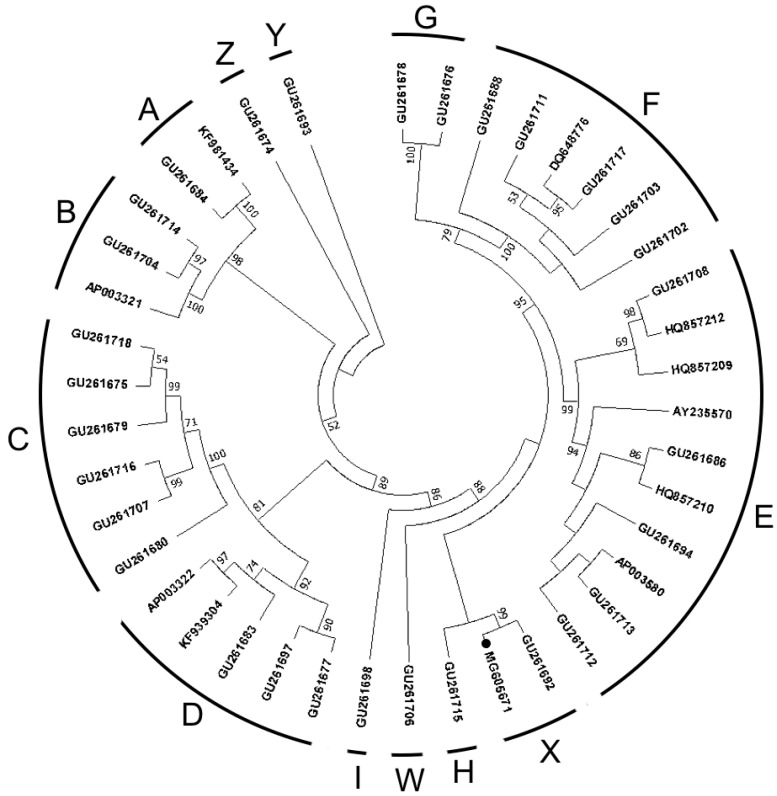
Phylogenetic tree of 41 complete mitochondrial genomes of *Gallus gallus* constructed with maximum parsimony.

A phylogenetic tree (Figure 1) of all known *Gallus gallus* mitogenomes was constructed and tested with 1 000 bootstrap replications using the MEGA 7 software package (Tamura et al., 2013). The tree showed that the Thai RJF mitogenome clustered with GU261692 from Yunnan (Miao et al., 2013) into haplogroup X, as defined by Miao et al. (2013) and Peng et al. (2015). To the best of our knowledge, all domestic chickens are distributed in haplogroups A to G. However, the arrangement of the RJF was identical to haplogroup X, which is only found in wild chickens. In the MP tree, all haplogroups had very high bootstrap values, all larger than 90%. The root of the MP tree was in haplogroup Y, according to the Chicken Reference Tree in dometree.org (Peng et al., 2015). Thus, our study strongly supported the previously defined reference tree. 

Black dot represents the new sample in this study. Bootstrap support values (>50) are shown at the nodes.

Here, we assembled the first complete mitogenome of the Thai RJF. This study will provide useful information for future evolutionary and population genetics analyses of the RJF and domestic chicken.
